# Assessing the Effectiveness of House-to-House Visits on Routine Oral Polio Immunization Completion and Tracking of Defaulters

**Published:** 2014-06

**Authors:** Dora Ward Curry, Henry B. Perry, Syed N. Tirmizi, Allison L. Goldstein, Meg C. Lynch

**Affiliations:** ^1^CARE-USA, 151 Ellis Street, NE, Atlanta, GA 30303, USA; ^2^Johns Hopkins Bloomberg School of Public Health, 615 N. Wolfe Street, Baltimore, MD 21205, USA; ^3^Centers for Disease Control and Prevention, 1600 Clifton Rd. Atlanta, GA 30333, USA; ^4^228 W. Lexington St., Baltimore, MD 21201-3443, USA

**Keywords:** Community health workers, Defaulter tracking, Home visits, Oral polio immunization, Polio eradication, Routine immunization

## Abstract

Strengthening routine immunization is one of the four prongs of the Global Polio Eradication Initiative. Using data collected through 30-cluster sample household surveys of caretakers of children aged 12-23 months, this paper assessed the effectiveness of house-to-house visits on routine oral polio immunization completion, using simple frequency tables, bivariate and multivariate logistic regression analyses. Logistic regression results demonstrated that children in households where the caregivers reported receiving a household visit by health workers were more likely to be fully immunized for polio through routine immunization than other children, although results were significant only after correcting for confounders. In Ethiopia and India, children of caregivers who remembered a house-to-house visit were significantly and positively associated with routine polio vaccination completion (OR=2.2 and OR=2.2 respectively). In Angola, the association was positive, though not significant (OR=1.3). The evidence suggests that targeting high-risk areas for house-to-house visits played a role in increasing routine polio vaccination.

## INTRODUCTION

The Global Polio Eradication Initiative (GPEI)is a collaboration of World Health Organization (WHO), national governments, Rotary International, the US Centers for Disease Control and Prevention (CDC), and the United Nations Children's Fund (UNICEF). Since 1988, the GPEI has implemented a four-pronged strategy across the globe to detect and stop the transmission of poliovirus through surveillance and immunization: acute flaccid paralysis (AFP) surveillance, oral polio immunization through the routine immunization system that provides vaccination for all antigens, supplementary polio immunization, and mop-up campaigns in which teams return immediately after a supplementary immunization campaign to areas shown to have inadequate coverage of oral polio vaccination (OPV). The CORE Group Polio Project (CGPP) has supported the objectives of the GPEI since 1999 when it began implementing (i) community-based AFP surveillance, (ii) support for OPV provision through the routine immunization system by promotion in the community and system strengthening for healthcare providers, and (iii) participation in supplementary immunization campaigns. The CGPP works only in high-risk areas as determined jointly with each country's Inter-agency Coordinating Committee, using objective criteria, such as routine immunization rates, campaign quality indicators, and polio transmission history. The CGPP is a USAID-funded project of the CORE Group, a membership organization of more than 50 US-based non-governmental organizations working in community-based maternal and child health. At the time of this evaluation, the CGPP was active in three countries (Angola, Ethiopia, and India), implementing activities in communities through 12 US-based NGOs, and 9 local NGOs, with World Vision acting as administrative host and CARE providing technical support at the global level. Funding available for the activities discussed here was up to 30 million US dollar over five years, distributed over these 21 NGOs in subgrants that ranged from approximately 50,000 US dollar to 300,000 per year per NGO. Allocations to NGOs were determined based on factors, such as local costs, population covered, and intensity of activities.

Strengthening routine immunization is a cornerstone of the GPEI (See Box for methods of vaccination in the GPEI). The GPEI aims to ensure high routine coverage of all OPVs in the national immunization schedules. Because of the poliovirus's ability to circulate undetected for long periods of time, immunization coverage must be quite high (80 to 85% in Africa and greater than 95% in Asia) even in subpopulations—not just at the national or provincial level—to interrupt transmission.

The CGPP uses a variety of social mobilization/communication strategies to promoting routine immunization and complementing mass media's social mobilization activities conducted by GPEI partners among hard-to-reach populations. A key element of the CGPP approach is to complement mass media's social mobilization activities conducted by partners, providing one-on-one interpersonal communication (IPC) visits to the households most at risk of missed vaccination. Community workers (paid a monetary stipend in some contexts, volunteers receiving only non-monetary rewards in others) maintain a list of children younger than five years of age in their catchment areas and visit the households to encourage the family to bring their children to receive OPV as well as other antigens at routine immunization opportunities. Community workers target families based on the level of risk determined, in part, by the immunization status of the children living in each household as well as by quality of routine services in the area and demographic risk factors.

**Box. Principles of immunization opportunities in Angola, Ethiopia, and India**

Routine Immunization–Offered in a fixed site (clinic or stationary booth at central location) by permanent staff of the Ministry of Health (MoH).

Campaign–Offered either door-to-door or at multiple booths in neighbourhoods with mobilizers bringing families to booths, sometimes, staffed by volunteers

Child Health Days in Angola and Ethiopia—Additional fixed-site booths offer OPV as well as other interventions with intensive social mobilization beforehand by permanent MoH staff

Mop-up Campaigns–Follow-up door-to-door vac-cination after a campaign, conducted by campaign personnel in areas identified by campaign evaluation to have been poorly covered

Unlike campaigns or most routine immunization systems, community health workers of CGPP visit houses (door-to-door) but do not offer vaccine

Household visits used an interactive counselling approach. Community health workers (CHWs) asked families about their reasons for not seeking vaccinations and responded with information appropriate to concerns of each family. Community health workers were prepared to respond to specific questions about the disease and the vaccine as well as to provide logistical and practical information.

Contents discussed in training and ongoing supportive supervision of community health workers had different emphases in each country, based on concerns of the local community. For example, in India, community health workers received training on how the vaccine was produced to re-assure families that the vaccine contained no ingredients that violated Muslim religious requirements in India where rumours to the contrary had spread. In Angola, by contrast, where demand was high but awareness of specific vaccination opportunities was low, contents focused on when and where the vaccine would be available while, in Ethiopia, contents addressed specific traditional beliefs about the spiritual rather than biomedical aetiology of paralysis. Community health workers also held health education activities. The timing and frequency of the visits ranged from 15 to 30 minutes, on average, with significant variation and occurred as often as every two months for families with children still needing routine vaccinations.

In India, the CGPP has an extensive network of 1,325 community mobilization coordinators (CMCs) who conduct social mobilization for polio vaccination through strategies that rely on direct personal communication with families individually and in small groups and in both informal and formal community settings (1). CMCs’ work in tracking the immunization status of all children younger than five years in all households in their catchment areas has been demonstrated to improve campaign coverage (1). CGPP Angola designed and implemented a child registration system in 2009, based on the successful CGPP India registry system. Using these registers, approximately 2,000 volunteers support tracking of immunization defaulters (children receiving one or two doses but not completing the three doses required to confer long-lasting immunity) in both poor urban and rural areas of Angola. In Ethiopia, the CGPP volunteers conduct IPC during home visits as well as in monthly meetings organized around traditional coffee ceremonies, a social gathering that naturally congregates people to discuss current issues (**2**). In all three countries, community health workers support supplemental immunization activities by conducting rallies and health education sessions prior to vaccination campaigns and by disseminating the dates of upcoming campaigns in their home visits.

This paper examines the effectiveness of house-to-house health promotion visits by lay health workers in increasing completion of at least three doses of OPV through the routine immunization system compared to residents of the intervention catchment area, who did not report receiving a household visit by health workers. Because both intervention areas and the households within the intervention areas were selected based on being at high risk for missed vaccinations and polio transmission, these findings may underestimate the effect of the intervention. These findings are based on analysis of household surveys conducted as part of a larger programme evaluation at year one and three of a five-year programme.

Over the years, the GPEI has successfully utilized a full range of social mobilization (SM) techniques, primarily focused on mass media campaigns and information dissemination approaches (television, radio and print ads, stickers, banners, vests, caps, flags, billboards, and songs with carefully-tested messages) (3). Data show that mass media campaigns, because of their potential to reach large numbers of people, have greatly contributed to the success of the GPEI (3). However, these are only effective if the type of media selected is accessible to the priority population (4-6). For example, a study by Waisbord (2004) in the D.R. Congo found that media campaign was only effective in providing information about NIDs in urban areas but, in most regions, television and radio had little impact (6). Further evidence demonstrates that heavy reliance on radio and television is effective in urban areas (7) but country reports consistently show that mass communication campaigns are minimally effective in reaching the poorest, the most marginalized people, and those with the most difficult access to health services (8). Thus, in the current emergency stage of the effort to eradicate polio, the GPEI has recognized that mass media campaigns are important for ensuring national visibility and raising awareness but have limited impact without the support of interpersonal communication (IPC) activities (3,6,7,9). According to a global assessment of communication strategies for polio by Waisbord (2004), the most effective information dissemination approaches require a mix of IPC and ‘mid-media’, or strategies aimed at a larger group than an individual or family but with a smaller reach than mass media (3,6,7,9) [Examples of mid-media are town criers, community meetings, messages at churches and mosques, cultural events, posters, and local radio]. In India, intensive and repeated IPC activities are conducted by cadres of trained health workers and communicators. Activities include house-to-house visits as well as systematic and sustained mobilization of community and religious leaders (3) supported by mid-media activities, such as wall writings, posters, and banners to create a visual presence (7-9). Similarly, following outbreaks in Niger, Nigeria, and Pakistan, IPC strategies were implemented to strengthen contact with the local community and local leaders to promote access to and ensure immunization of all children in the area (10).

In marginalized communities where routine immunization is weak and vaccination is still not a social norm, IPC strategies are used as a “persuasion tactic”, effectively shifting people's knowledge, attitudes, and beliefs about OPV and, thus, changing immunization practices (3,6,7,9,10). Evaluation studies have unanimously supported these findings, concluding that IPC provides the most culturally- and linguistically-appropriate communication channels, particularly in rural areas without access to mass media (5).

The effectiveness of CHW interventions targeting immunization has been documented in numerous reviews. In a review of the published literature about interventions aimed at increasing immunization coverage in developing countries, Pegurri *et al*. found that, while most interventions reported an increase in the percentage of fully-vaccinated children (FVC), with a mean increase in coverage of 27% (11), CHW interventions and door-to-door canvassing strategies resulted in the greatest increase in the proportion of FVC (12). Likewise, a review of case studies in India and Pakistan by Obregón and Waisbord (2010) found that the number of OPV refusals and polio cases significantly decreased in areas where CHWs worked (8).

The GPEI frequently uses house-to-house tracking methods to increase immunization by filling the gaps in the formal health service (7). For example, in Ecuador, health workers in Amazon River community were hired to identify hard-to-ﬁnd households and enhance delivery of services (12). India has been using similar techniques to reach rural communities and strengthen routine immunization services since 1999. These techniques involve training of CMCs to conduct house-to-house visits and inform people about polio immunization by visiting, engaging, and mobilizing families and caregivers (7,12).

A review by Ryman (2008) provides numerous global examples of GPEI programmes that utilize house-to-house visit strategies. In Ghana, both non-health workers and health workers visited homes of defaulting children, leading to an increase in the percentage of FVC from 60% to 85% in the intervention group compared to 61% to 67% in the control group. In Mexico, household visits by lay workers increased the percentage of FVC among those for children less than one year of age from 21% to 77% in five months compared to the control group where coverage minimally increased from 30% to 35% (4). Lastly, a study conducted by Maekawa (2007) in Lao demonstrated that house visits significantly influenced the rate of fully-immunized children among mothers living in areas with poor routine immunization services (13).

With local variations in each country, the CGPP uses maps and/or registers of complete immunization history to indicate which households to be prioritized for home visits. Similar strategies have been successful in Bangladesh and South Africa where community workers carried out targeted home visits. During the intervention, 87% of children in Bangladesh referred by these volunteers completed the recommended immunization series in an observational study while 67% of children in the South African intervention had completed their third dose of OPV by eight months of age in comparison with 50% of the non-intervention group (4).

Although house-to-house tracking by using detailed community maps is a standard approach for campaigns, it is rarely used for routine immunization. The CGPP employs this technique to improve OPV coverage through the routine immunization system. In all three countries under study, community workers maintain detailed lists of households, denoting number of children younger than five years of age, name of head of household, and location. Through different mechanisms depending on the country, the community workers have access to each child's immunization status. In Ethiopia where most community workers are not fully literate, they coordinate closely with the Health Extension Workers of the Ministry of Health, who maintain registers of individual children with their immunization histories, to identify non- or partially-immunized children for visits. In both Angola and India, the CGPP community workers maintain their own similar immunization registries tracking OPV doses both from routine system sources and during supplementary immunization activities.

Community workers plan their household visits based on the risk of under-immunization. Households with newborns and those with children overdue for a vaccination are the highest priority. In addition, households with pregnant women, those with women close to term in their pregnancies, and those in communities that have exhibited high levels of resistance (due to cultural barriers or access to health services) have also high priority. Finally, if feasible, a community worker may also visit a household with a child scheduled to receive a routine immunization dose in the near future. The CGPP also maintains maps of the area of each community workers, with individual households designated on the map either by a tracking number or by the same numbering system used on the corresponding register, again, either at the local health post level or by the community workers themselves.

The use of high-risk status to target both intervention areas and the households visited may have affected the findings of this analysis. Because the intervention areas were selected based on high risk (using factors, such as low estimates of routine immunization and history of polio transmission) and households within the intervention area selected to receive visits based on high risk (using factors, such as the family having refused vaccination or belonging to an underserved ethnic or religious group or a child there being overdue for routine vaccination), these results likely underestimate the impact of house-to-house visits. On the other hand, it is possible, though unlikely, that households visited by community health workers were more accessible to both community health workers and routine immunization services. In that case, these results could overestimate the effect of this technique.

## MATERIALS AND METHODS

### Data collection

In Ethiopia, India, and Angola, trained interviewers administered the questionnaire. A sample based on the WHO's 30-cluster sampling methodology was selected in each country. Respondents were mothers/caretakers of children aged 12-23 months. In Ethiopia, a complete 30-cluster sample with 10 respondents in each cluster was drawn from each of the three project areas, covering seven zones and representing agrarian, pastoralist, and semi-pastoralist populations, for a total sample of 900 respondents in August 2010. In India, a complete 30-cluster sample with 10 respondents in each cluster was drawn from two project areas in Uttar Pradesh, representing urban and rural populations for a total sample of 600 in July 2010. In Angola, a single 30-cluster sample covering the entire project area across five provinces, with 15 respondents per cluster, was drawn for a total sample of 450 respondents in August 2010. The surveys were conducted by local data-collection firms external to the project. The surveys from which the data presented here were drawn took place as the second of the three periodic surveys for programme evaluation. Limitations in the dataset for the first survey conducted in 2008 made comparisons between 2008 and 2010 data impossible and, at the time of submission of this paper, data from the final survey conducted in 2012 were not yet available.

The survey instrument was adapted principally from the DHS immunization and demographic modules (MEASURE DHS, 2008). The researchers also developed additional questions to capture respondents’ attitudes about and knowledge of acute flaccid paralysis (AFP), vaccination, home visits, and other activities of community workers. The survey instrument was translated into local languages, back-translated into English to verify accuracy, and then field-tested in each country.

### Analysis

In all three countries, MS Excel was used for entering the mid-term evaluation survey data, which were transferred to SPSS datasets and cleaned. Independent variables included were: socioeconomic status; geographic area; and mothers’ knowledge, attitudes, and perceptions concerning vaccinations; and exposure to the intervention as defined by respondent's recall. The dichotomous definition of the dependent variable categorized children as fully immunized for polio through the routine system or not fully immunized. Immunization status was determined first from the child's immunization card. In cases where a card was not available, the mother's recall of her child's immunization status was used. Mother's recall was used in determining immunization status for 47%, 63%, and 45% of the responses in Angola, Ethiopia, and India respectively. A child was considered fully immunized against polio through the routine system if he/she had received three or more OPV doses as part of the routine vaccination system (i.e. not during a supplementary immunization activity). Interviewers recorded information referring to the oldest child aged 12 to 23 months living in the household. Simple frequency tables, bivariate and multiple logistic regression results were obtained using SPSS (version 15.1).

## RESULTS

Table 1 presents the rate of full routine immunization coverage against polio in CGPP area in each country. In Ethiopia, 75.8% of children had received at least three routine doses of oral polio vaccine (OPV) compared to 69.2% in India and 61.8% in Angola.

### Socioeconomic characteristics

In Ethiopia, the majority (57.1%) of respondents were non-literate. One half (50%) spoke their own area's main dialect rather than other dialects. The majority were long-term residents, suggesting low mobility among majority of the population in the project area. A significant minority (38%) reported working outside the home. The majority lived within 30 minutes walking distance from a health facility/clinic (Table 2).

In India, majority of the respondents in the project area were non-literate and mostly long-term residents. Almost all respondents (99%) spoke the main language. A small percentage (3.8%) of mothers of young children worked outside the home. The majority (72.2%) lived within 30 minutes walking distance from a health facility.

In Angola, 28.4% of the respondents in the project area were non-literate. Slightly over half (56.2%) spoke Portuguese. Most respondents (64.4%) reported working mainly outside the home. Most (66.7%) reported being long-term residents. Most (57.1%) lived within 30 minutes walking distance from a health facility.

**Table 1.** Child polio immunization status by country CGPP project area

Polio immunization status of child	Ethiopia N (%)	India N (%)	Angola N (%)
None/Partially immunized	201 (24.2)	186 (30.8)	172 (38.2)
Fully immunized	629 (75.8)	417 (69.2)	278 (61.8)

**Table 2.** Sociodemographic characteristics

Characteristics	Ethiopia	India	Angola
	Total	Total	Total
	N (%)	N (%)	N (%)
Literacy status			
Non-literate	474 (57.1)	393 (65.2)	128 (28.4)
Literate	356 (42.9)	210 (34.8)	322 (71.6)
Language spoken			
Main dialect	416 (50.1)	597 (99.0)	253 (56.2)
Other dialects	414 (49.9)	6 (1.0)	197 (43.8)
Length of stay at current residence			
>1 year	42 (5.1)	245 (40.6)	151 (33.6)
1 to <11 years	254 (30.6)	358 (59.4)	299 (66.4)
≥11 years	534 (64.3)	−	−
Mother works outside home			
No	518 (62.4)	580 (96.2)	160 (35.6)
Yes	312 (37.6)	23 (3.8)	290 (64.4)
Distance to health post			
30 minutes or more	232 (28.0)	32 (5.3)	193 (42.9)
<30 minutes	598 (72.0)	571 (94.7)	257 (57.1)
Type of area			
Pastoralist/rural	263 (31.7)	300 (49.8)	255 (56.7)
Semi-pastoralist/urban	281 (33.9)	303 (50.2)	195 (43.3)
Agrarian	286 (34.5)	−	−

### Bivariate analysis

Table 3 presents the child immunization coverage levels stratified by geographic area and whether the child's caregiver received a community worker's visit. Across all geographic project areas and in all three countries, with the exception of rural Angola, the rate of child polio immunization completion among households that had been visited by a community worker was higher than that among households that had not been visited. Coverage rates in visited households in Ethiopia were 88.3%, 79.4%, and 82.2% in pastoralist, semi-pastoralist, and agrarian regions compared to 59.5%, 74.8%, and 76.4% respectively in non-visited households. Completion rates in India in visited households were 76.9% and 75.9% in rural and urban areas compared to 70.7% and 76.4% respectively in non-visited households. Completion rates in Angola in visited households were 55.7% and 76.2% in rural and urban areas respectively compared to 55.6% and 66.7% in non-visited households.

### Multivariate analysis

Using logistic regression, after controlling for potentially confounding variables, including caregiver's literacy, length of stay at residence, urban or rural residence, and whether the caregiver worked outside the home, full vaccination of children with OPV through the routine system was significantly and positively associated with caregivers’ recalling receiving a visit from a community worker in Ethiopia and India (OR=2.2 in both countries). In Angola, the association was positive but not statistically significant (OR=1.3).

Due to the non-significant results in Angola, the authors performed additional analysis on the urban/rural differences in the coverage. Considering the results of a three-way cross-tabulation comparing urban and rural vaccination rates further stratified by whether the household recalled having received community worker's visit, the difference between urban and rural polio vaccination completion was statistically significant, regardless of the household visit.

**Table 3.** Polio immunization status by type of area and community worker's home visit

Type of area	Immunization status	Community worker's home visit					
		Ethiopia		India		Angola	
		No	Yes	No	Yes	No	Yes
		N (%)	N (%)	N (%)	N (%)	N (%)	N (%)
Pastoralist/rural	Fully immunized	94 (59.5)	88 (88.3)	157 (70.7)	60 (76.9)	69 (55.6)	73 (55.7)
Semi-pastoralist/urban	Fully immunized	116 (74.8)	100 (79.4)	112 (59.9)	88 (75.9)	88 (66.7)	48 (76.2)
Agrarian	Fully immunized	55 (76.4)	176 (82.2)	-	-	-	-

In Ethiopia, completed polio vaccination was significantly and positively associated with caregivers’ knowing the correct timing of the first dose of polio vaccination (referring to the dose immediately after birth known as OPV0) (OR=3.2), speaking the main dialect of the area (OR=2.7), and working outside the home (OR=1.9). Caregivers’ having resided at their present home for one to 10 year(s) was significantly and negatively associated (OR=0.1) with children having completed routine polio immunization (in contrast to those whose caregivers had resided at the current location for less than one year (Table 4).

In India, completed polio vaccination was significantly and positively associated with caregivers’ knowing the correct timing of the first dose of polio vaccination (OPV0) (OR=1.9) and being literate (OR=2.0).

In Angola, completed polio vaccination was significantly and positively associated with caregivers’ knowing the correct timing of the first dose of polio vaccination (OPV0) (OR=1.5), living in an urban area (OR=2.1), and being literate (OR=1.9). Complete polio vaccination was significantly and negatively associated with caregivers’ working outside the home (OR=0.5).

Project areas in Ethiopia cannot be exactly categorized as rural or urban, unlike in India or Angola. Thus, contextual variables, such as length of stay and work outside the home are not directly comparable. The Hosmer and Lem show goodness-of-fit test for the multivariate regression results across Ethiopia, India, and Angola, which shows that the model fits the data well (Table 4).

## DISCUSSION

The evidence presented supports the conclusion that the CGPP approach of targeting high-risk areas for house-to-house visits played a role in increasing routine OPV3 completion. The child of a caregiver who reported a visit by a health worker to the home was more likely to have completed the routine polio immunization series in all three countries, even after controlling for potential confounding variables. The effect-size was strong, and the association was statistically significant in India and Ethiopia. The association observed in Angola was not significant but was positive.

The non-significant results in Angola may represent the mitigating role of physical access to services. In Angola, 43% of caregivers lived within more than 30 minutes walking distance from a health facility, distinctly more than in any other country in the programme (28% in Ethiopia and only 5% in India). In rural areas of Angola, 56% of children had completed their routine polio vaccination regardless of whether they had been visited by a lay health worker. In urban Angola, among caregivers who did not report a visit by a health worker, 56% of children had completed their routine polio vaccination while 67% of children whose caregiver did not report a visit had done so. Since a difference in vaccination completion was observed between visited and non-visited households in urban areas, but not in rural areas, the non-significant results may be due to the larger rural population (57%), masking the effect of the intervention. The significant difference between rural and urban areas in Angola for vaccination completion, controlling for the effect of community worker's home visit rates, strengthen this interpretation.

**Table 4.** Logistic regression results for routine immunization (RI) status—cross-country comparison

Variable	Ethiopia		India		Angola	
	Exp (β) Odds ratio	95% CI	Exp (β) Odds ratio	95% CI	Exp (β) Odds ratio	95% CI
Community worker's visit						
Did not remember (reference)						
Remember the visit	2.22*	1.21-4.08	2.21*	1.26-3.88	1.26	0.82-1.94
Region						
Pastoralist (reference)						
Semi-pastoralist	1.04	0.44-2.49	−	−	−	−
Agrarian	1.81	0.75-4.39	0.75	0.47-1.22	2.05*	1.20-3.51
Literacy						
Non-literate (reference)						
Literate	0.94	0.52-1.71	2.01*	1.15-3.50	1.83*	1.14-2.94
Language						
Other dialects (reference)						
Main dialect	2.73*	1.33-5.60	−		0.66	0.39-1.11
Length of stay in residence						
0 to <1 year (reference)						
1 (1-10) years	0.10*	0.01-0.82	−	−	−	−
2 (11 or less) years	0.18	0.02-1.401	1.27	0.76-2.15	1.43	0.93-2.22
Mother works outside home						
No (reference)						
Yes	1.89**	1.00-3.57	−		0.55*	0.35-0.86
Walking distance to health post						
<30 minutes (reference)						
30 minutes or more	0.92	0.44-1.94	0.72	0.24-2.17	0.88	0.58-1.34
Goodness-of-fit Hosmer and Lem show test p value for χ^2^	0.918*	−	0.750*	−	0.312*	−
Cox and Snell R^2^	10.4%	−	7.6%	−	8.8%	−
Nagelkreke R^2^	18.0%	−	11.0%	−	12.0%	−

*Significant at p<0.05;

**Not significant at p<0.10

Examination of the immunization status in rural and urban areas further stratified by distance from a health centre revealed a differential impact of distance from facility on immunization status. In urban areas, 67% of the children of respondents living within more than 30 minutes from the nearest health facility were fully immunized while 73% of those living within 30 minutes walking distance from their facility had completed their third dose of OPV. In rural areas, the proportion of those completing their routine polio vaccination, who lived within 30 minutes walking distance or further from their facility, was similar to urban areas at 63% but, among rural residents living within more than 30 minutes walking distance from the facility, only 46% were fully immunized. This differential impact provides evidence that, in rural areas, CGPP volunteers may have been effective in improving caregivers’ receptivity to vaccination but are incapable of overcoming the barrier of geographic distance.

The unexpected negative association between length of stay in residence in Ethiopia may be related to rural/urban dynamics as well. In the other two settings with dense, mobile urbanized populations, a short length of stay may have been associated with less integration into the community and less familiarity with how to access social services. In the more rural setting in Ethiopia, however, short length of stay may have been associated with having moved closer to a town where health services were provided while more length of stay may have been associated with a more geographically remote location farther from routine immunization services.

Another unexpected finding was the positive association between the caregiver working outside the home in Ethiopia and complete vaccination, paired with a negative association with the same two variables in Angola. Work outside the home in the intervention areas of rural Ethiopia may be paired with greater exposure to towns where health posts are located and, therefore, more with greater access to vaccination services. In Angola, on the other hand, work outside the home may be associated with street vending or selling in the market, where work schedules make visiting the health post during the day difficult.

The knowledge of the importance of the first dose was also observed to be predictive of routine OPV completion. These results were statistically significant in all three countries. While this model treats this knowledge as an independent variable, the positive association between a house-to-house visit and vaccination completion suggests that a partial mechanism for the effect of house-to-house visitation is through increased understanding of how the polio vaccine works and how to access it. Within the CGPP, even in populations where resistance has been a significant problem, delivery of specific knowledge about the polio virus and vaccine, validated by locally recognized authorities, such as religious leaders, has been a key part of strategy of the project.

The full CGPP addresses larger issues, like social acceptability simultaneously with knowledge gaps. One distinctive feature of CGPP activities is that they reach individual households that may not have received information through other services about routine OPV immunization.

### Limitations

The principal limitation of this study is its cross-sectional design. Similar results from a pre- and post-intervention and comparison group design would further strengthen the evidence base. In addition, the home visit was implemented in the CGPP context as part of a package of several activities, including community announcements, mid-media small group education talks, and mass communication. Without further evaluation and stronger study design, it is impossible to determine with certainty the relative role of house-to-house visits as distinct from the role of other interventions. Also, measurement of visit was based on the respondents’ recall, and no data were available to validate the accuracy of that recall.

The challenge of replicating this study in a truly comparable population is significant due to the elevated risk profile of the populations in the study area. Each country selected all of its programme areas specifically because the children were at the highest risk of missed vaccination. Using a matrix of objective criteria, including routine immunization coverage with pentavalent vaccine, coverage in previous polio vaccination campaigns and history of poliovirus transmission, the official polio eradication body in each country designates high-risk areas, and within each assigned high-risk area, the CGPP targeted the most underserved populations. The characteristics of the populations covered in this study are, therefore, extremely hard to match.

The impact of wider community mobilization efforts is also difficult to exclude when attempting to estimate the impact of these activities. In these settings, other efforts via channels, such as mass media and the health system, are significant and have some impact on communities’ and families’ likelihood to seek and accept vaccination. The inability to control for exposure to other social mobilization efforts for polio was a limitation of this study.

### Conclusions

Polio eradication efforts and other programmes for the hardest-to-reach populations should consider utilizing the strategy of house-to-house visits by lay health workers. Developing a clearly-articulated risk profile and identifying the highest-risk social groups or geographic areas for top priority to receive visits may be helpful. As a specific example of the considerable variation in risk factors between countries, caregivers who worked outside the home were more likely to have completely vaccinated children in Ethiopia despite the fact that, in India and Angola, the opposite was true. Again, in contrast to the other two countries in this analysis, in Ethiopia, a mother recently arriving at her place of residence was more rather than less likely to have completely vaccinated children. In India and Angola, both a longer time of residence in the current location, resulting in greater familiarity with health services available and freedom from the demands on her time predicted a mother's greater ability to access vaccination services for her children. In contrast, more recent arrival in current home location and work outside the home predicted greater uptake of vaccination services among their children in Ethiopia, perhaps because mothers who had recently moved or worked outside the home were more likely to have access to vaccination services offered in more urbanized areas. These unexpected findings and the variation in these findings by country illustrate the importance of determining a risk profile based on data specific to the intervention area. The CGPP plans to continue to refine its risk profiles to identify social and geographic factors influencing subpopulations at risk of under-immunization. Refined risk profiles will allow targeting interventions to account for subnational variations, such as whether the caregiver works outside the home or speaks the main dialect/language of the area as appropriate to the district.

Since house-to-house visits can be an effective mechanism to reach the hardest-to-reach subpopulations, future programming may benefit from house-to-house visitation for promotion of routine immunization to families or populations least likely to access the service on their own. Programmes implementing integrated health or other elements of health promotion could explore the utility of house-to-house visits in those settings. House-to-house visitation is indicated for all families (prioritized according to the child's individual age and immunization status) within CGPP catchment areas because the programme works only in settings already defined as high-risk areas. For a programme covering the general population, this intervention would seem to be most effective when directed at those areas or social groups most likely to undervalue or resist immunization. Another important consideration when determining whether to include house-to-house visitation in a community worker health programme is the ease of impacting the health behaviour in question as well as such considerations as paid or volunteer status, other duties assigned to the community health workers, and the availability of resources for training and ongoing support for them.

One implication of these results is that the impact observed was on routine polio immunization coverage rather than campaign polio immunization status. These results contribute to the argument that polio eradication has had some beneficial effects on related routine immunization. Because of the imperative to identify and gain access to every child younger than five years, the CGPP and other partners in the Global Polio Eradication Initiative have developed new ways not only to provide the hardest-to-reach populations with information but also to ensure they receive services.

The usefulness of household visitation as a strategy to improving coverage of routine polio vaccination, one of the four pillars of the global polio eradication strategy, has not been documented previously. The results of this study suggest that household visits are an important tool in supporting routine immunization. Household visits can improve routine polio immunization coverage in the hardest-to-reach areas that are especially at-risk to be unreached by traditional clinic-based activities. At a time when the success of the GPEI hangs in the balance and every possible advantage is required to provide polio vaccination to children in the most inaccessible households, the private volunteer organizations (PVOs) and NGOs involved in the CGPP and others working at the community level can utilize household visits and other community-based approaches to making a key contribution to the final push to the eradication of polio.
